# Old-Growth Forests and Bryophyte Communities in Italy and the Broader Mediterranean Region: A Literature Review

**DOI:** 10.3390/plants14182824

**Published:** 2025-09-10

**Authors:** Mattia Letizia Marino, Patrizia Campisi, Fortunato Cirlincione

**Affiliations:** 1 Department of Agricultural, Food and Forest Sciences, University of Palermo, Viale delle Scienze, Bldg. 5, I-90128 Palermo, Italy; 2 Department of Biological, Chemical and Pharmaceutical Sciences and Technologies, University of Palermo, Via Archirafi 38, I-90123 Palermo, Italy; patrizia.campisi@unipa.it; 3 National Biodiversity Future Center (NBFC), University of Palermo, Piazza Marina 61 (c/o Palazzo Steri), I-90133 Palermo, Italy; 4 Department of Soil, Plant and Food Science (Di.S.S.P.A.), University of Bari “Aldo Moro”, Campus “E. Quagliarello”, Via Edoardo Orabona 4, I-70125 Bari, Italy; fortunato.cirlincione@uniba.it

**Keywords:** forest naturalness, ecological continuity, mosses, liverworts, old trees, deadwood, biodiversity conservation, indicator species

## Abstract

Beginning with general references to old-growth forests and the numerous benefits that they provide at multiple levels, this review mentions the main surveys conducted in Italy to identify and characterise Italian old-growth forests and offers an overview of the state of knowledge on bryophytes of these ecosystems in Sicily. Then, it focuses on the relationship between bryophyte diversity and old-growth traits (e.g., structural characteristics, long-term continuity), as well as the potential use of bryophytes as bioindicators of forest continuity and naturalness. In this regard, studies on bryophyte floras and communities in old-growth forests were examined in detail not only for Italy but also for the broader Mediterranean region, also taking into account evidence from investigations conducted in other bioclimatic zones. The analysis shows that old-growth forests often provide refuges for rare and noteworthy taxa and host highly diverse bryophyte communities. However, it appears that in Mediterranean forests, which have been less studied than temperate and boreal forests, the influence of certain factors that are known to be important in other contexts, such as deadwood, may be comparatively less relevant. Also, bryophyte species highly related to old-growth stands or with mature and ancient trees in the Mediterranean area are reported.

## 1. Introduction

Old-growth forests have attracted increasing attention since the end of the last century, as reflected by the substantial rise in related research activities [1]. Indeed, the awareness of the importance of such ecosystems of high naturalness has grown in a world more and more affected by human impact [2].

Research conducted across diverse geographical regions, climatic conditions, and forest types has demonstrated that old-growth forests exhibit unique ecological, structural, and biological characteristics compared to more disturbed or managed forests of the same type [3,4].

Notably, studies on cryptogamic communities in these relatively undisturbed ecosystems are of particular interest, as they may help to shed light on the distinctive traits of old-growth forests and enhance understanding of their ecological uniqueness [5].

This paper aims to synthesise research on the ecological and structural peculiarities of old-growth forest ecosystems, with a particular focus on Italy and Sicily, one of its major islands, as well as to present data from investigations on the influence of forest old-growthness on bryophyte floras and communities. These studies are summarised and analysed to highlight consistent and recurrent results, address unresolved questions, and identify potential gaps in current knowledge.

### 1.1. The Significance of Bryophytes in Forest Environments

Within forest ecosystems, bryophyte communities are usually highly diverse, representing a considerable proportion of overall species richness. Beyond their taxonomic importance, they act as ecosystem engineers, exerting a wide range of ecological functions that influence forest dynamics and the provision of ecosystem services [6].

From a functional perspective, bryophytes contribute to soil formation processes, accelerating pedogenesis. They are also involved in hydrological regulation: given their high water-holding capacity, they are able to absorb water in a short time and gradually release it, thereby reducing soil evaporation and maintaining a more stable and humid microclimate at the forest floor [6,7].

In addition, they play an active role in biogeochemical cycles: several bryophytes contribute to nitrogen fixation, while their capacity to capture and retain nutrients prevents the leaching of essential elements, enhancing nutrient availability within the forest ecosystem [6].

Bryophytes are also key pioneer organisms, particularly effective in recolonising open or disturbed substrates, such as those left bare after fires or treefall events. By covering the soil surface, they stabilise the substrate, limit erosion phenomena, and create more suitable conditions for the establishment of other organisms. Finally, bryophyte carpets affect vascular plant diversity and regeneration: they may facilitate seedling establishment by trapping seeds, or, conversely, inhibit growth through allelopathic effects and light reduction [6,7].

### 1.2. Old-Growth Forests: Definition and Value

Many terms with slightly different meanings have been used to describe old-growth forests [8].

With reference to Italy, the most recent definition has been provided in Art. 3(2)(s-bis) of the “Consolidated text on forests and forestry supply chains” approved by Legislative Decree No. 34 of 3 April 2018. Specifically, they are issued as “wooded areas made up of native species consistent with the biogeographical context, characterised by a peculiar biodiversity deriving from the absence of disturbance for at least sixty years and by the presence of successional stages of development linked to spontaneous regeneration and senescence”.

At the international level, another notable definition is that adopted by the Convention on Biological Diversity (CBD), which refers to old-growth forests as “stands in primary or secondary forests that have developed the structures and species normally associated with old primary forest of that type have sufficiently accumulated to act as a forest ecosystem distinct from any younger age class” [9].

From these descriptions, it can be inferred that old-growth forests are late-successional forest ecosystems that have not experienced significant anthropogenic disturbances over extended periods and that, even when originating from secondary forests, have regained the natural complexity that renders them similar to old primary forests.

Old-growth forests play a crucial role in mitigating climate change by providing shaded, sheltered environments. Their structural complexity—characterised by dense biomass and tall canopies—buffers temperature increases and creates favourable conditions for species sensitive to warming [10].

Beyond this, they deliver key ecosystem services. In this respect, aside from their role as oxygen producers, old-growth forests function as significant carbon sinks, accounting for at least 10% of global carbon sequestration capacity [11]. Old trees store a great amount of carbon and contribute to its sequestration by also creating suitable conditions for its retention in the soil [12].

Furthermore, old-growth forests moderate local climate through their capacity to regulate hydrological cycles. In particular, they enhance precipitation and reduce the frequency of dry and hot days, thereby exerting a positive influence on human health [13].

More broadly, they contribute to human well-being in multiple ways. For example, they host fungi that serve as sources of medicinal compounds, such as the agarikon mushroom *Fomitopsis officinalis* (Vill.) Bondartsev & Singer [14], *Laetiporus sulphureus* (Bull.) Murrill, and *Pseudoinonotus dryadeus* (Pers.) T. Wagner & M. Fisch [15]. Moreover, owing to their lower burn rates, intact forests can act as natural barriers, helping to mitigate the impact of fires on human settlements [13].

Old-growth forests are also an integral part of the cultural heritage of human populations worldwide, providing significant socio-cultural benefits [14].

They likewise represent a key resource for forest management, offering unique opportunities to study natural forest dynamics, serving as essential benchmarks for silvicultural practices that emulate natural processes [1]. This sustainable approach is increasingly necessary, since relatively undisturbed forests have proven to be more resilient and more effective in delivering ecosystem services than altered forests [13].

### 1.3. Old-Growth Forests in Italy

Despite their importance, undisturbed forest ecosystems are currently very rare, as most forests worldwide have undergone substantial alterations over millennia, often cleared for pastures or agricultural land, or exploited for timber used in construction and fuel [16]. Such exploitation has been particularly pronounced in regions with historically high levels of human activity, including Europe and the Mediterranean [17,18]. This helps to explain why primary or old-growth forests in Europe today are limited and fragmented, accounting for only about 3% of the continent’s total forest cover [8].

Consequently, it is unsurprising that the conservation of old-growth forests has become one of the main priorities of environmental policies [19]. The European Plant Conservation Strategy (2008–2014) already highlighted their value, particularly considering their high vulnerability in southeastern Europe [20]. More recently, the EU Biodiversity Strategy for 2030 and the New EU Forest Strategy for 2030 have underscored the importance of formally recognising these ecosystems and call for strict protection of all remaining primary and old-growth forests within the EU [21,22].

Italy has translated these European commitments into action through the National Biodiversity Strategy (Ministerial Decree No. 252 of 3 August 2023), aiming to ensure rigorous protection of all primary and old-growth forests in the country. Italy’s attention to its forest heritage, however, predates these initiatives. The country had already approved the “Consolidated Text on Forests and Forestry Supply Chains”, which led to guidelines for identifying areas that can be classified as old-growth forests, as well as for their management and protection (Ministerial Decree No. 608943 of 19 November 2021).

These guidelines also aimed to establish a National Network of Italian Old-growth Forests, which was subsequently formalised by Interministerial Decree No. 0193945 of 5 April 2023. The establishment of this extensive national database, designed to safeguard the diversity of flora and fauna of Italy’s old-growth forests, has spurred research initiatives focused on their inventory and characterisation [23].

Until the early 2000s, in Italy, the presence of forests exhibiting old-growth characteristics was largely unknown and considered unlikely, given pervasive forest exploitation dating back to Roman times [24]. However, studies conducted in recent decades have identified several forests with old-growth features across the country, e.g., [19,25,26,27].

A first comprehensive investigation identified 68 forests in Italy’s Natural Parks with old-growth traits [28]. The stands were selected based on structural features—such as the diameter of living trees, the quantity of deadwood, and the stage of decay—and assigned to one of three old-growthness categories (high, medium, or low) according to a cumulative score.

To date, a total of 166 stands with old-growth traits have been recorded in Italy and, as reported by Motta [1], the vast majority of them are characterised by the dominance of beech (*Fagus sylvatica* L.), sometimes also in association with silver fir (*Abies alba* Mill.). In addition, among the Italian beech stands with high ecological value, those that have been included in the list of “ancient and primeval beech forests of the Carpathians and other regions of Europe” recognised by UNESCO as World Heritage Sites are certainly worth mentioning [29].

However, in Italy, where forest resources have been intensely and continuously exploited, some compromises have been necessary to ensure that a sufficient number of suitable stands are included in the old-growth inventory and protected [1]. For example, as noted above, the “Consolidated Text on Forests and Forestry Supply Chains” defines old-growth forests as those that have not experienced anthropogenic disturbances for at least sixty years. Although sixty years may seem adequate, scientific knowledge accumulated over time suggests that this period is likely insufficient for the development of the structural characteristics typical of old-growth forests [1].

### 1.4. Old-Growth Forests in Sicily

In Sicily, initial studies highlighting the importance of certain old-growth stands date back over twenty years [30,31] and subsequent contributions focused on the inventory of individual monumental trees [32,33].

Over the past decade, research has increasingly focused on the identification, valorisation, and protection of old-growth forest ecosystems. Investigations in Sicily have concentrated on stands exhibiting old-growth traits, analysing their distribution, floristic composition, structural attributes, and ecological functions. Such studies are intended to generate knowledge that can inform conservation strategies, monitoring protocols, and assessments of biodiversity and forest continuity [34,35,36,37,38,39,40,41,42,43].

As a result, a set of stands with distinctive features has been identified, 22 of which have recently been nominated for inclusion in the Italian Network of old-growth forests. These stands, listed in Table 1, are mainly located in Sicily’s major mountain ranges, within regional parks, nature reserves, and sites of the regional Natura 2000 Network [35].

The criteria taken into account to identify the stands were: the abundance of old trees; tree species richness; the degree of naturalness of both shrubs and herbaceous layers; the structural complexity; the presence of a high amount of dead biomass; the richness of habitats; the possible presence of traces of human activity [35].

**Figure 1:**
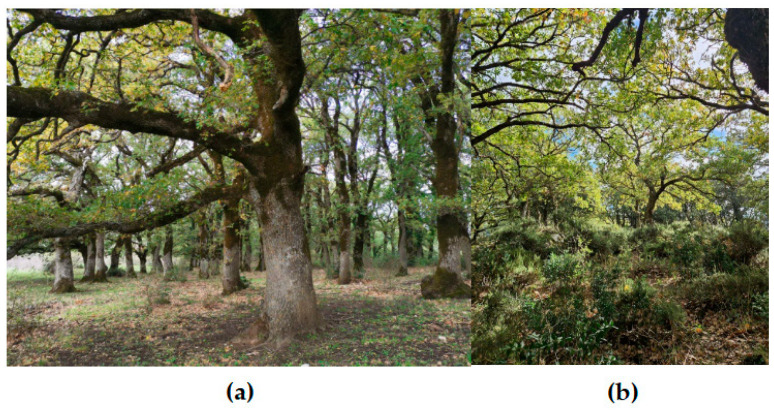
(**a**) An overview of Bosco del Fanuso, a deciduous oak forest of Sicily exhibiting old-growth traits dominated by the downy oak; (**b**) vegetation layers of Bosco del Fanuso.

As reported in Table 1, the forest types exhibiting old-growth characteristics in Sicily primarily include beech and oak forests, both evergreen (holm oak and cork oak forests) and deciduous (Turkey oak, sessile oak, and downy oak forests) (Figure 1).

Additionally, *Pinus nigra* subsp. *calabrica* forests and other stands characterised by the presence of *Ilex aquifolium* L. or *Taxus baccata* L. trees have also been identified. This represents a significant number of undisturbed forest ecosystems. Indeed, Sicily is one of the Italian regions with the highest number of sites displaying old-growth traits, second only to Abruzzo [1]. Moreover, this number is likely to increase in the future, as ongoing research may identify additional stands with similar characteristics.

In this context, a recent proposal suggested including a residual strip of sclerophyllous forest—unaffected by human impact for several decades—among the Sicilian forests with old-growth traits. This forest is located within the Favorita Urban Park in Palermo and forms part of the Monte Pellegrino Oriented Nature Reserve. Investigations at this site have revealed communities and taxa typical of mature forest formations, some of which are unusual at this altitude, encompassing both vascular and non-vascular plants, as well as fungi [45].

Some forests identified in Sicily, however, do not fully meet the criteria to be classified as old-growth. For example, the above-mentioned guidelines (Ministerial Decree No. 608943 of 19 November 2021) specify that forests must have a minimum area of 10 hectares to ensure the maintenance of the natural ecological processes typical of a mature forest. Yet, as shown in Table 1, several Sicilian forests are smaller than this threshold. These forests may still qualify as old-growth, since the guidelines further state that “in exceptional cases, specifically justified by particular characteristics, the minimum area may be reduced to 2 hectares, provided that the area still constitutes a single ecological-site system, both functional and structural (…); regions may also approve provisions for the identification and protection of plant formations that are consistent with the old-growth characteristics (…) but which do not meet the above-mentioned area requirements, identifying them as islands of senescence (…)”.

Aside from size, another limitation is that Sicilian forests are not entirely free from human disturbance, as they remain partially subject to grazing [35].

## 2. Results and Discussion

### 2.1. Bryophytes in Old-Growth Forests

In line with the objectives of this study, we summarised the main findings concerning the relationship between bryophytes and old-growth forests. Overall, the research conducted so far highlights that these ecosystems are particularly significant for bryophyte communities.

Indeed, numerous studies have shown that they generally host bryophyte communities characterised by higher species richness, e.g., [46,47,48]. They also contain a greater proportion of rare and red-listed taxa [49,50], including species with habitat or substrate specialisation that restrict them exclusively to these environments [51,52].

It has been observed that bryophytes are particularly sensitive to forest alteration, e.g., [47,53,54,55,56], more than other organisms (such as vascular plants) [57,58]. Human disturbance leads to losses in terms of number of species within this taxonomic group, with a particularly negative impact on conservation of relevant species [59]. This sensitivity is likely due to the poikilohydric nature of these organisms [60]. As bryophytes are unable to prevent desiccation, directly relying on the surrounding environment for water supply, they are highly dependent on environmental factors and vulnerable to their fluctuations [61]. In this regard, forest harvest affects bryophyte communities by determining modifications on ecological conditions, since canopy disruption reduces moisture and increases light [62].

However, there are also studies in which, when comparing unmanaged with managed forests, greater species richness has been found in the latter [63,64], in accordance with the intermediate disturbance hypothesis [65]. This hypothesis assumes that moderate disturbance does not necessarily harm biodiversity but may, on the contrary, promote it, as it alters the integrity of the ecosystem and creates new microhabitats that can be colonised by species that previously lacked suitable ecological conditions for establishment (e.g., light-demanding species).

Nevertheless, some authors emphasised that even in cases where managed forests exhibit a greater number of bryophyte species, the conservation value of such areas is not equivalent to that of undisturbed forests. In fact, the bryophyte communities of old-growth forests are dominated by specialist bryophyte species and the bryophyte floras include a higher number of rare taxa, while disturbed forests tend to be dominated by common generalist species [59,66,67].

Among bryophytes, liverworts appear to be particularly affected by human pressure [67], as these organisms require higher levels of humidity compared to mosses, having life forms that make them very susceptible to drought and desiccation [68]. In fact, old-growth forests often host a significantly higher number of liverwort taxa than disturbed forest ecosystems since they create a more humid and shady environment [46,69,70].

The importance of the particular microclimatic conditions provided by semi-natural forests for bryophytes has been highlighted, for instance, by a study carried out by Cooper-Ellis [51] in western Massachusetts (USA). The study pointed out that old-growth forests hosted twice the number of epiphytic bryophyte species than second-growth forests, even when the sampled trees were of the same species and had the same diameter. The difference in species richness was primarily attributed to the distinctive ecological conditions that characterised old-growth forests, including high moisture, dimmed light, and specific bark features.

Beyond this, other important traits distinguish old-growth forests from younger or disturbed forests.

Among these characteristics, long-term forest continuity (i.e., the continuous presence of forest cover at a given location over long periods of time) is certainly a factor that can positively influence bryophyte diversity [71]. In particular, it is crucial for species with limited dispersal abilities, which are organisms that generally reproduce asexually or produce spores infrequently. In this case, long-term continuity plays a major role since it provides the time necessary to dispersal-limited species to colonise the habitat and to establish [53,54,72,73]. Although some studies have stressed that age is not always the main limiting factor for bryophytes [74], it undoubtedly gains particular significance in contexts where the landscape has been markedly altered by human activity, as in Europe. In such cases, high fragmentation increases distances, with negative repercussions on dispersal-limited species [69].

Furthermore, long-term forest continuity has an indirect effect on other processes. For example, it allows time for stochastic events to occur within the ecosystem. These phenomena may help to explain part of the bryophyte diversity observed in a specific place. Indeed, some studies support the idea that community composition is also determined by a succession of random establishment processes, causing differences between sites that are otherwise similar with reference to environmental variables or other inherent factors [74,75].

Another distinctive feature of old-growth forests concerns their remarkable structural complexity, which results in an increase in microhabitat availability. In fact, in such forest ecosystems, the structural dynamics, generated by the natural forest development, tend to favour the species richness of forest-dwelling bryophytes as these processes increase spatial heterogeneity of resources like light, water, and nutrients [50,74,76,77]. For example, the death of single old trees leads to gap formation, creating openings and increasing light penetration [78]. Also, tree mortality due to uprooting, which is an event much more frequent in old-growth forests than in younger stands, generates new substrates by exposing roots in the tip-up mound, as well as rocks and bare soil, which can be colonised by less competitive species [79,80]. The higher degree of niche partitioning can explain why, in old-growth forest ecosystems, β-diversity (species turnover) is generally higher compared to secondary forests [69], even in cases where α-diversity (species richness) is similar [81].

**Figure 2:**
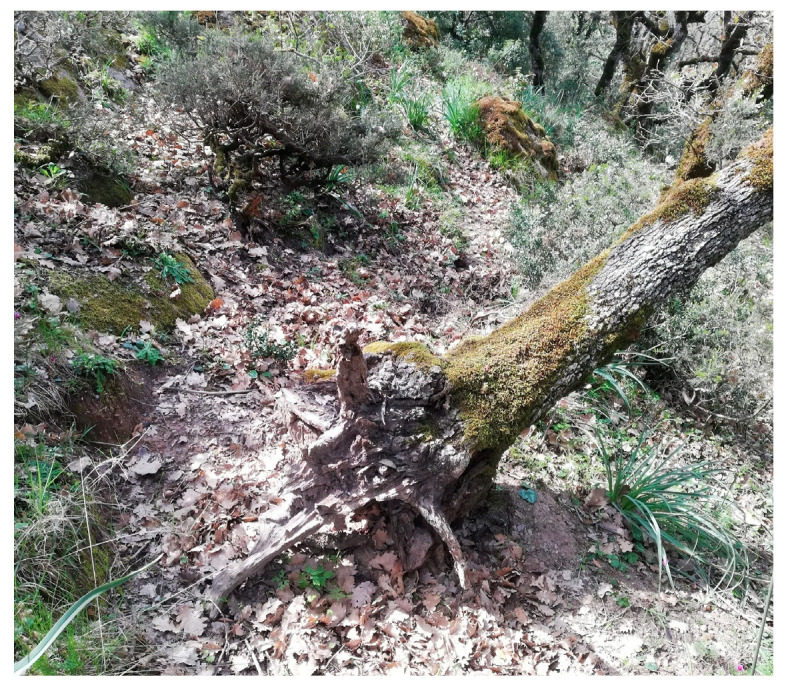
Bryophyte communities on a fallen tree. Dead, lying trees represent one of the forms in which deadwood occurs in forests and constitute important ecological legacies for taxa with high specialisation for this substrate.

With reference to the structural complexity of old-growth forests, and particularly to the availability of different substrates, it is important to mention the presence of deadwood. In fact, compared to younger or managed forests, old-growth forests exhibit a greater abundance and variety of this substrate, providing numerous microhabitats that support the establishment and persistence of many bryophyte species (Figure 2), including epixylic specialist taxa [5,53,54,82,83,84,85,86].

These microhabitats arise from the diverse physical and environmental conditions offered by decaying wood, such as different levels of moisture, temperature, and light exposure. Ecological conditions change, in fact, depending on the characteristics of deadwood units, which can differ in size (coarse woody debris, fine woody debris), type (logs, stumps, snags), orientation (horizontal or vertical), and decomposition degree (freshly fallen or well-decayed wood) [87].

Specifically, large-sized pieces of deadwood have proven to be particularly important for bryophytes compared to smaller fragments [5,85]. The significance of large-sized units is certainly related to the increased available surface area for colonisation that they offer, but also, and more importantly, to the temporary nature of deadwood [46,53]. In fact, since large deadwood legacies require longer periods to decompose, they provide the time for more species to establish and reproduce [53,82,88].

Indeed, the decomposition process of deadwood creates a dynamic environment that fosters a wide range of ecological niches for epiphytic and epixylic bryophytes, until the wood develops into a substrate indistinguishable from the forest litter and becomes suitable for the establishment of ground species [89].

Regarding the type of deadwood, many studies highlighted that logs appear to be the ideal substrate for the colonisation of mosses and especially liverworts, as they have higher moisture-retention capacity [46,53,54,83,86]. Also, stumps can be important for maintaining higher levels of bryophyte diversity in forest ecosystems [82,86].

With reference to the progression of the decomposition process, bryophytes seem to reach their maximum richness in early decay stages on horizontal substrates, such as logs [71,86]. Higher diversity on vertical substrates, like dead standing trees, has occasionally been noted in later stages [71]. Species turnover is also observed, as the composition gradually shifts from early to late decay classes, in accordance with the changes in substrate characteristics due to decomposition [46,54,89]. In fact, freshly fallen deadwood essentially hosts intact epiphyte communities that had already developed on living trees. As the bark detaches and the wood gradually loses its original consistency, these epiphytes are replaced by epixylic species. Finally, when deadwood is in an advanced state of decomposition and nearly converted into humus, the ground species take over and achieve dominance [46,54,89].

The total amount of deadwood in a forest can also be a significant factor affecting the survival of epixylic taxa. Since deadwood does not persist over time, its continuous renewal is crucial to sustain bryophyte populations. In fact, high availability of this substrate enables species to disperse from a disappearing patch to newly suitable sites for colonisation [53].

The tree species from which the deadwood originates represents another important variable influencing bryophyte diversity [86,90].

Other structures primarily found in old-growth forests are the large, old trees, which provide unique substrates for bryophytes [49,91]. Old trees represent different habitats compared to younger trees, even when they belong to the same species, as with ageing, the bark undergoes both chemical and structural changes, such as variations in pH, texture, water retention capacity, and roughness [51].

In this respect, many epiphytic bryophytes exhibit a preference for structured bark, a substrate that is age-dependent since bark becomes thicker and more deeply fissured as trees mature [58]. Given that bryophytes typically require high moisture levels, this preference can be understood in light of the increased water retention within bark fissures, which benefits these organisms [92]. Additionally, it has been suggested that the fissures in the bark have a positive effect on bryophytes by providing favourable conditions for germination [93]. Also, variations in bark pH can affect bryophyte communities, determining associations with specific host tree species or age classes, thereby influencing the spatial patterns of epiphytic taxa [94].

As for the longevity of old trees, it has a positive effect on bryophyte communities by providing a stable and reliable substrate, which is particularly important for all those taxa that require longer periods of time available for colonisation, such as the aforementioned dispersal limited species [94].

Moreover, old trees are usually large-sized and therefore provide a greater bark surface area compared with younger trees. Such a characteristic can explain a significant part of bryophyte species richness when the limiting factor is space rather than time [49].

**Figure 3:**
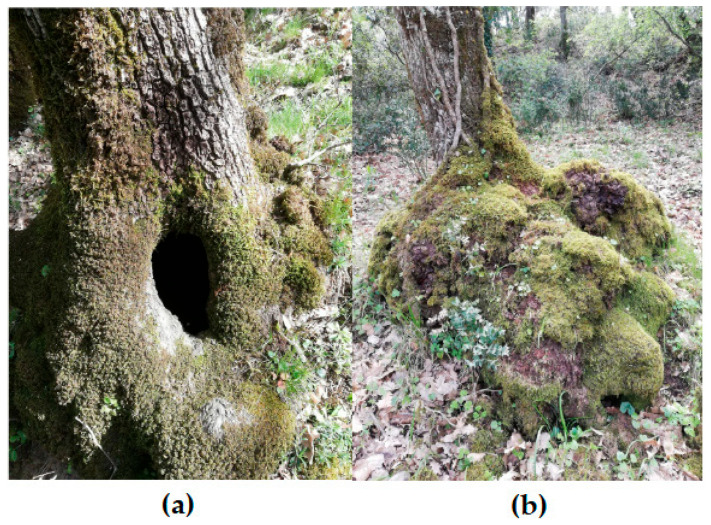
(**a**) A rot hole, a tree-related microhabitat commonly found on senescent trees, which supports bryophyte diversity by creating an environment characterised by high moisture and acidity; (**b**) Excrescences, another tree-related microhabitat associated with old trees, which constitute a peculiar substrate for bryophyte species.

Another important aspect of senescent and damaged trees is the presence of distinctive formations, such as excrescences or cavities (Figure 3) [95]. These structures act as specialised microhabitats for bryophyte species, some of which are highly specific [96].

For instance, the epiphytic moss *Codonoblepharon forsteri* (Dicks.) Goffinet, a species rare in Sicily that is threatened both at the national and European level, exclusively grows inside and on the edges of tree holes, where rainwater and leaves accumulate, or in fissures where acidic water percolates from such cavities [97].

### 2.2. Bryophytes as Indicators of Ecological Continuity and Forest Naturalness

A key aspect of bryophytes in relatively undisturbed forests lies in their pronounced sensitivity to disturbance regimes, which affect forest age, structure, and related environmental variables [98]. Since several species are predominantly restricted to these ecosystems, they are widely used as indicators of old-growth forests, providing scientific support for the identification of wooded areas of conservation interest [99,100,101].

Indeed, among bryophytes, species with limited dispersal abilities, such as the moss *Neckera pennata* Hedw., have been recognised as good indicators of forest naturalness [102]. Similarly, specialist epiphytic or epixylic species that grow on old trees and deadwood have also demonstrated a strong association with old-growth forests, making them valuable indicators of long-term forest continuity [103]. Moreover, the richness of species of conservation concern, including red-listed taxa, has proven to be a reliable indicator of forest integrity, as such organisms are more affected by human impact [59,104]. However, although species richness and general abundance of specialist and rare taxa can be successfully used as informative parameters [4], in some contexts, the composition of bryophyte communities shows an even more sensitive response to disturbance processes, making it a more effective indicator of forest naturalness [105].

Furthermore, the frequency or abundance of species displaying specific functional traits, particularly those related to life forms and life history strategies, can also provide valuable insights into long-term habitat continuity and degree of naturalness in forest ecosystems [73,106]. In this regard, bryophytes classified as “perennial stayers”, which are well-suited to stable environmental conditions (i.e., some pleurocarpous mosses), have been proposed as possible indicators of ecological continuity [73]. Nevertheless, the potential and reliability of these indicators have been scarcely investigated, as most studies did not consider a functional approach [73].

However, it has to be underlined that the use of biological indicators has its limitations, as species identified as indicators in a specific geographic region or climatic zone may not occur in another region or may exhibit a different behaviour, thus losing the ability to provide useful information on the surrounding environment [107].

### 2.3. Bryophytes in Old-Growth Forest Ecosystems of Italian Territory and in the Other Countries of the Mediterranean Area

According to the results of this research, current knowledge on the bryophytes of old-growth forests across the Mediterranean area is still very limited.

For instance, one of the few available studies is that of Sabovljevic et al. [46], conducted in the Central Balkans (Serbia and Montenegro), which compared the diversity of saproxylic bryophytes in an old-growth beech forest with that in a managed beech forest. The study emphasised the importance of undisturbed forest ecosystems, and specifically of the presence of deadwood in different stages of decay, for the conservation of epixylic bryophytes. In fact, the results showed that the old-growth stand fostered higher diversity of these taxa and hosted a greater number of threatened and rare species. Also, the study has underlined that saproxylic specialist taxa can potentially be used as indicators of old-growth forest.

Another relevant research addressing the relationship between bryophytes and old-growthness in the Mediterranean region has been conducted in the subtropical laurel forest of Madeira, resulting in the identification of a set of seventeen taxa significantly associated with undisturbed forests [108]. The list included four slow-disperser species that are endemic to Madeira, which are *Andoa berthelotiana* (Mont.) Ochyra, *Frullania polysticta* Linbemb., *Heteroscyphus denticulatus* (Mitt.) Schiffn., and *Plagiochila maderensis* Gottsche ex Steph.

Regarding the Italian territory, only a few studies have examined the bryophyte floras of these environments with reference to stand age or specific structural parameters. At the national level, the only research addressing these issues are those by Brunialti et al. [109] and Blasi et al. [76], both focused on Turkey oak and beech stands in the Cilento, Valle di Diano, and Alburni National Park (Campania).

These studies provide interesting insights into forest dynamics in the Mediterranean area, which may differ from processes observed in other regions. For instance, deadwood may play a less prominent role in Mediterranean environments. Brunialti et al. [109] highlighted the lack of correlation between the amount of deadwood and bryophyte community diversity, interpreting this as a consequence of faster wood biodegradation under the higher temperatures typical of Mediterranean forests, which reduces the time available for bryophyte colonisation. According to this study, the structural factors that most notably influenced bryophytes were the presence of old trees, a broad range of tree diameter classes, high levels of tree basal area, and high understory diversity.

However, the role of deadwood in Mediterranean forests warrants further investigation. Blasi et al. [76], examining the diversity of multiple taxonomic groups in relation to various old-growth structural attributes, found that deadwood occurrence could indeed be important. This study showed that only the species richness of groups directly associated with forest woody components, including bryophytes, was significantly linked to old-growth forest structure. Moreover, bryophyte species richness was clearly related to structural complexity, further underscoring the importance of microhabitat availability for these small organisms.

As regards investigations focused on the identification and use of indicator species or parameters of long forest continuity, although Italy hosts a relatively high number of studies on lichens and saproxylic insects, similar research on bryophytes remains very limited [1].

Specifically, only a study by Puglisi et al. [110] and the previously mentioned studies by Brunialti et al. [109] and Blasi et al. [76], all conducted at the same location, address this topic. Puglisi et al. [110] applied bryovegetational analysis based on biological and ecological traits (e.g., life forms, life strategies, or sensitivity) to distinguish areas subject to strong human impact from those exhibiting high naturalness and air quality.

Brunialti et al. [109] highlighted that the mosses *Homalothecium sericeum* (Hedw.) Schimp. and *Antitrichia curtipendula* (Hedw.) Brid., previously regarded as indicators of long forest continuity in other regions, could also serve as potential indicators in the Mediterranean area, as they were consistently associated with the old-growth stands considered in the study, regardless of forest type.

Regarding the outcomes of Blasi et al. [76], certain bryophyte species, such as *Leucodon sciuroides* (Hedw.) Schwägr. and *Isothecium alopecuroides* (Lam. ex Dubois), when occurring alongside other species from different taxonomic groups, can serve as indicators, thereby facilitating the identification and monitoring of old-growth forests.

**Figure 4:**
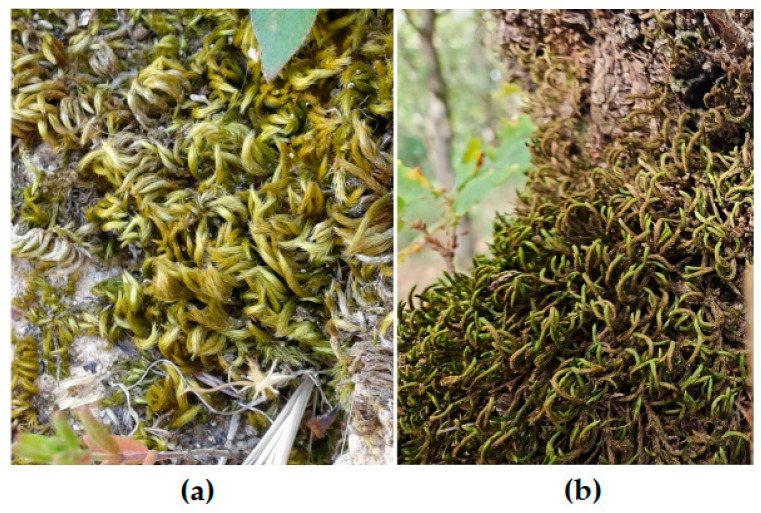
(**a**) The moss *Homalothecium sericeum* (Hedw.) Schimp., a species regarded as an indicator of long-term forest continuity across different biogeographic regions, which may also serve this role in the Mediterranean area based on current knowledge; (**b**) *Leucodon sciuroides* (Hedw.) Schwägr., a moss that achieves high frequency and cover in bryophyte communities of old trees in Mediterranean forests, emerging as a possible indicator of old-growth characteristics in these ecosystems.

Overall, these findings appear to align with the few observations made in Sicily related to such ecosystems, which included references to some of these species. For instance, in the sessile oak forest of Pomieri, located in the Madonie Mountains (province of Palermo), a coenosis dominated by *Leucodon sciuroides* (Figure 4) and characterised by high frequency and cover values for *Homalothecium sericeum* (Figure 4), was found on the ancient trees [30].

Furthermore, a study conducted on individual holm oak trees of various ages at Piano Zucchi, near the Pomieri forest, detected similar epiphytic communities while investigating bryophyte succession from twigs to old trees [111].

Notably, the pleurocarp *Leptodon smithii* (Hedw.) F. Weber & D. Mohr dominated the communities on older trees, while *Leucodon sciuroides*, present only on trees with diameters exceeding 20 cm, also played a significant role, becoming the dominant species in the absence of *Leptodon smithii*. The species *Homalothecium sericeum* was found exclusively on old trees, suggesting a possible association, although it did not achieve dominance. In a similar study from Spain on *Quercus pyrenaica* Willd. trees, the results were comparable: on older trees, *Homalothecium sericeum* had increased cover values, and *Leucodon sciuroides* was dominant, alongside the pleurocarpous moss *Pterigynandrum filiforme* Hedw. [112]. In both studies, bryophyte succession involved the gradual replacement of communities dominated by drought-tolerant acrocarpous species with communities dominated by pleurocarpous species. These latter communities appear to constitute a micro-climax of bryophyte vegetation, typical of the stable ecological conditions found in old-growth forests [30].

The aforementioned Bosco di Pomieri, in the Madonie Regional Park, as well as the Nature Reserve of Bosco di Malabotta, in eastern Sicily, are the only two forests exhibiting old-growth traits of the island subject to specific bryofloristic studies [30,113,114].

These investigations had already partly revealed the considerable interest of the bryophyte component of the flora. They included rare and threatened taxa such as *Fissidens bryoides* Hedw. var. *cespitans* Schimp. and *Grimmia meridionalis* (Müll.Hal.) E.Maier, both endangered in Italy and reported for Pomieri [115]. Another notable species is *Grimmia fuscolutea* Hook., recorded in Bosco di Malabotta, which is considered Endangered in Italy and Vulnerable at the European level [115,116]. Furthermore, some individual reports relating to the presence of bryophyte taxa in other forests of Sicily with old-growth features can be found in studies not specifically aimed at these wooded areas [106,117,118,119,120,121].

Overall, on the basis of the analysis conducted, fragmentary knowledge on bryophytes of the old-growth Sicilian forests has been compiled and a list of taxa known to date has been assembled.

These are 183 specific and infraspecific taxa, including 155 mosses and 28 liverworts, distributed across the forest areas for which data are available in the literature. A comprehensive dataset is provided in the Supplementary Materials (Table S1), which reports both the distribution of these taxa among the investigated forests and their IUCN conservation categories, as listed in the Italian and European red lists [115,116].

As can be seen in Table 2, this is a complex of taxa with high taxonomic diversity, including 51 families belonging to 101 genera. The most represented family is *Brachytheciaceae*, followed by *Pottiaceae* and *Orthotrichaceae*.

A small group of taxa among those recorded so far in these Sicilian environments is of particular conservation interest, as they are considered rare and threatened.

In particular, in addition to the three rare moss species already mentioned for the Pomieri and Malabotta sites, five species are classified as Near Threatened, four at the national level (the liverwort *Riccia macrocarpa* Levier and the mosses *Grimmia decipiens* (Schultz) Lindb., *Lewinskya shawii* (Wilson) F. Lara, Garilleti & Goffinet and *Tortula canescens* Mont.), and one at the European level (the moss *Ceratodon conicus* (Hampe) Lindb.).

**Figure 5:**
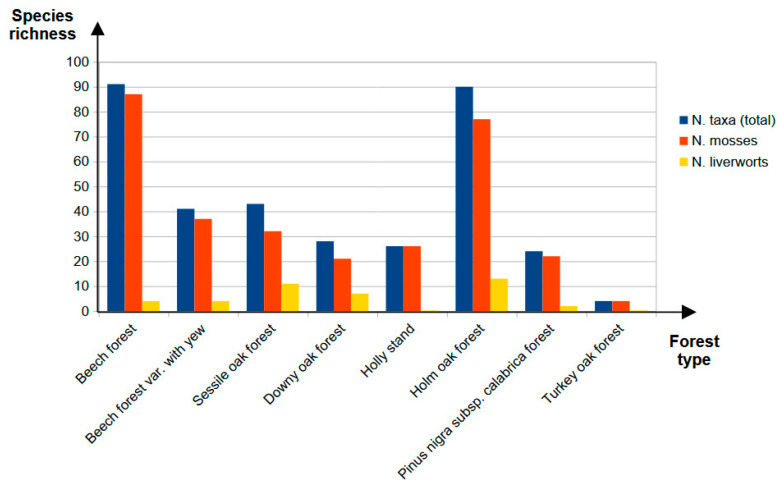
Distribution of species richness across the different forest types of Sicilian stands proposed as old-growth.

With reference to the distribution of taxa across the different forest types of Sicilian stands with old-growth characteristics, an overview is provided in Figure 5, shown both as total taxa and with mosses and liverworts considered separately.

The graph includes only forest types for which data on bryophyte floras were available, grouped and synthesised as described in the Section 4.

The figure shows that beech forests host the highest total species richness compared to other forest types (91 taxa). This highlights their importance as habitats for bryophytes, although part of this richness may be attributable to the fact that a large proportion of the selected stands are beech–dominated forests, which has allowed the collection of more related information. By contrast, it is noteworthy that holm oak forests also display remarkably high species richness (90 taxa), with values comparable to those of beech forests, despite covering a considerably smaller area than the latter. The species richness of the sessile oak forest (43 taxa) is also significant, given the limited extent of the single stand considered, even though it falls within intermediate values together with downy oak forests (28 taxa), the holly stand (26 taxa), and the forests characterised by the presence of yew (41 taxa). The lowest number of species was found in the *Pinus nigra* subsp. *calabrica* forests (24 taxa) and, in particular, in the Turkey oak forests (with only 4 taxa). The apparent paucity of species in the latter forest type is undoubtedly due to the fact that it has so far been only marginally studied, thereby highlighting the existence of a knowledge gap.

When examining the distribution of bryophyte groups across different forest types, it emerges that holm oak-dominated forests, in addition to hosting a large contingent of mosses, also contain the highest number of liverworts (77 mosses, 13 liverworts). In beech forests, by contrast, mosses account for the vast majority of species, while liverworts are represented by only a few taxa (87 mosses, 4 liverworts). Beech forests with yew, however, differ in this respect: this particular forest type hosts a proportion of liverworts approximately twice as high as that found in the other beech forests, thereby reducing the imbalance between mosses and liverworts (with 37 mosses, 4 liverworts). Overall, however, Sicilian beech forests appear to play a relatively minor role in the conservation of liverworts compared to other forest types. In this regard, it is interesting to note that a substantial number of liverworts are found in the sessile oak forest, where this group, comparable in consistency to that found in holm oak forests, accounts for about one quarter of the total taxa (with 32 mosses, 11 liverworts). In downy oak forests, too, the proportion of liverworts is considerably high, reaching one-third of the total (21 mosses, 7 liverworts). In other forest types, specifically those dominated by holly and the Turkey oak forests, only mosses have so far been recorded, with their numbers corresponding to the total taxa observed. Further investigations may reveal that these environments could also be suitable for liverwort colonisation.

## 3. Conclusions

The current knowledge on the old-growth forest heritage in Italy and Sicily highlights significant progress over the last twenty years in the identification and protection of these areas and their peculiarities, previously widely underestimated and poorly investigated.

With reference to bryophytes, the analysis of primary investigations carried out worldwide to study the influence of forest continuity on floras and communities revealed a general positive relationship between the diversity of these small organisms and old-growth traits. Overall, old-growth forests appear to be particularly favourable to bryophytes primarily due to long-term habitat continuity and structural complexity. Habitat persistence over time is the key factor for dispersal-limited species, while higher diversity in taxa with effective dispersal ability is likely driven by the greater variety of microhabitats. In particular, the presence of large, old trees and a greater availability of deadwood seem to have the strongest influence on bryophyte species richness and composition.

Regarding the use of bryophytes as indicators of ecological continuity and forest naturalness, most studies suggest taking into account taxa with limited dispersal ability, species highly dependent on deadwood, and specialist or rare taxa, including those listed in national and international red lists. The presence of red-listed species in Italian old-growth habitats further emphasises the conservation relevance of these ecosystems and underscores the need to monitor such taxa as priority targets for biodiversity protection.

From a management perspective, the conservation of bryophyte diversity in old-growth forests calls for specific measures. Among the most effective are the retention of deadwood in different decay stages, the safeguarding of large and senescent trees, and the limitation of practices that may alter microhabitats, such as excessive grazing or unsustainable forest interventions. These actions not only support bryophyte assemblages but also contribute to the broader integrity and resilience of old-growth ecosystems.

Bryophyte species richness and composition remain the most commonly analysed response variables to assess conservation value, temporal continuity, and degree of naturalness of forest stands. In contrast, functional traits of bryophytes, such as those linked to life history strategies, are still rarely considered and should be given more attention in future research, as they may provide valuable insights into ecosystem functioning.

This review also underscores that data on bryophytes in old-growth forest ecosystems of the Mediterranean region remain limited. Most studies refer to temperate and boreal zones, while only a few have been conducted in Mediterranean countries, and these often focus on a restricted range of forest types. As a result, knowledge gaps persist for many characteristic Mediterranean forest ecosystems—such as thermophilous downy oak or Turkey oak forests.

Research priorities should therefore include the following: (i) expanding bryophyte surveys across a wider range of Mediterranean forest types, (ii) integrating functional and ecological traits into monitoring frameworks, and (iii) clarifying how the interactions between bryophytes and forest structure may differ from patterns described in better-studied regions.

Strengthening research on bryophytes in Mediterranean old-growth forests is essential not only for advancing ecological knowledge but also for informing effective conservation strategies. In this sense, bryophytes should be fully recognised as key allies in identifying, managing, and safeguarding the last remnants of old-growth ecosystems in Italy and beyond.

## 4. Materials and Methods

The papers mentioned in this review were selected by accessing different sources. In particular, the Scopus, Web of Science, and Google Scholar were consulted.

Several Boolean algorithms were tested, involving numerous combinations of terms, in order to identify the most effective search strategy (i.e., the algorithms that retrieved all relevant results using the minimum number of words).

Specifically, the following string was used to find the investigations conducted to acquire information on Italian and Sicilian old-growth forests (“old-growth forest*” AND Italy).

As for the studies regarding the relationship between bryophyte diversity and well-preserved forest ecosystems, the following Boolean algorithm was launched on the platforms: ((“old-growth forest*” OR “old tree*” OR “old-growth wood*” OR “primary forest*” OR “ancient forest*” OR “virgin forest*” or “primeval forest*” OR “old-growth stand*” OR “monumental tree*”) AND (bryophyte* OR moss* OR liverwort*)).

In the Scopus database, the search was conducted in “Article title, abstract, keywords” while in the Web of Science database in “Topic”. Filters concerning the manuscript and source types as well as the language were applied to refine the search. Specifically, only English-language articles published in journals were considered, while no time constraints were used for the year of publication. The literature review was grounded on the results of primary studies, with journals selected as the main source type, given their higher reliability due to the rigorous peer-review process. Preference was given to research outputs published in English, as these offer broader accessibility. However, if other relevant studies were found to have been excluded due to the filtering criteria, they were subsequently incorporated to ensure comprehensiveness. This applied particularly to different document types, such as reviews, book chapters, or conference papers, when they provided pertinent information. In addition, the search concerning the Italian context was extended to include studies written in Italian.

The records obtained on this basis were first screened by titles and abstracts and subsequently examined in full to identify only those papers genuinely pertinent to the topic. This included research comparing forest stands with respect to age, management, and disturbance regimes. Studies were excluded if the comparisons did not involve stands that had experienced a sufficiently long period without disturbance (at least sixty years). For example, this was the case in investigations comparing forests subjected to different management practices.

Overall, the collection of information regarding the current state of knowledge on old-growth forests in Italy was limited to an overview of the main investigations and a summary of the key features of the woods, while for Sicily all available studies were taken into account.

With regard to bryophytes, in order to obtain a comprehensive picture of the impact of old-growth attributes on their diversity, studies from all investigated geographical and climatic regions worldwide, as well as from different forest types, were included and examined. Particular attention was devoted to the limited number of studies concerning Italy, Sicily, and the whole Mediterranean area (here considered as delimited by Ros et al. [122]), each of which were analysed and separately discussed.

As regards nomenclature of taxa, Aleffi et al. [123] and Bartolucci et al. [124] were followed for bryophytes and vascular plants, respectively.

Forest types of Sicily took into account the classification reported by Camerano et al. [125]. In Figure 5, forest types were indicated in a more concise manner, identifying each stand by its dominant tree species and by grouping them, so as to provide a clearer overview of the distribution of taxa. In this regard, the downy oak forest types (downy oak forest on calcareous substrates; xerophilous downy oak forest on calcareous substrates) were grouped together, as well as the holm oak forests (typical montane holm oak forest; submontane mesoxerophilous holm oak forest with holly), the Turkey oak forests (Turkey oak forest on mesoxeric soils with holly; montane Turkey oak forest; typical Turkey oak forest on mesoxeric soils), and the *Pinus nigra* subsp. *calabrica* forests (typical montane *Pinus nigra* subsp. *calabrica* forest, downy oak forest on siliceous substrates, trembling aspen stand; upper *Pinus nigra* subsp. calabrica forest, trembling aspen stand, downy oak forest on siliceous substrates; typical montane *Pinus nigra* subsp. *calabrica* forest; lower *Pinus nigra* subsp. *calabrica* forest). Similarly, beech forest types (calciphilous mesoxerophilous beech forest; typical montane mesothermal beech forest; mesophilous beech forest on siliceous substrates; beech forest on lava substrates of Etna) were also grouped together. However, it was considered appropriate to keep separate the beech forest type characterised by the occurrence of yew (forests of other native broad-leaved species, mesophilous beech forest on siliceous substrates var. with yew), given that the presence of this species distinguishes it from the other beech forest types, resulting in a non-negligible variability in the associated bryophyte communities.

The detailed information on the old-growth forests of Sicily presented in Table 1 was retrieved from the documentation available on the institutional website of the region of Sicily [44].

## Figures and Tables

**Table 1:** Overview of forests proposed for inclusion in the Italian Network of old-growth forests [44].

Location	Forest Type	Surface Area (ha)	Coordinates
*Quercus pubescens* Willd. s.l. forest of Santa Maria del Bosco	Downy oak forest on calcareous substrates/Xerophilous downy oak forest on calcareous substrates	12.15	37°41′50.09″ N 13°11′10.33″ E
*Quercus pubescens* Willd. s.l. forest of Fanuso	Downy oak forest on calcareous substrates/Xerophilous downy oak forest on calcareous substrates	11.91	37°51′26.67″ N 13°25′45.09″ E
*Ilex aquifolium* L. stand of Piano Pomo	Holly stand	2.84	37°53′45.348″ N 14°03′57.473″ E
*Fagus sylvatica* L., *Ilex aquifolium* L. and *Quercus petraea* subsp. *austrotyrrhenica* Brullo, Guarino & Siracusa forest of Piano Pomo	Calciphilous mesoxerophilous beech forest	10.80	37°53′45.348″ N 14°03′57.473″ E
*Fagus sylvatica* L. forest of Cozzo Luminario	Typical montane mesothermal beech forest/Calciphilous mesoxerophilous beech forest	2.11	37°53′59.495″ N 14°03′33.879″ E
*Quercus ilex* L. forest of Monticelli	Typical montane holm oak forest/Submontane mesoxerophilous holm oak forest with holly	10.38	37°54′35.145″ N 14°04′1.460″ E
*Quercus ilex* L. forest of Orippotto	Typical montane holm oak forest/Submontane mesoxerophilous holm oak forest with holly	31.14	37°54′16.926″ N 14°0′8.810″ E
*Quercus petraea* subsp. *austrotyrrhenica* Brullo, Guarino & Siracusa forest of Pomieri	Sessile oak forest var. with beech	6.81	37°51′44.779″ N 14°04′24.246″ E
*Fagus sylvatica* L. forest of Favarotta	Typical montane mesothermal beech forest/Mesophilous beech forest on siliceous substrates	6.96	37°54′45.940″ N 14°31′36.618″ E
Bosco della Tassita	Typical montane mesothermal beech forest/Forests of other native broad-leaved species, mesophilous beech forest on siliceous substrates var. with yew (*Taxus baccata* L.)	22.59	37°53′54.334″ N 14°28′49.786″ E
*Quercus cerris* L. forest of Sant’Antonio	Turkey oak forest on mesoxeric soils with holly/Montane Turkey oak forest	29.94	37°53′19.664″ N 14°32′22.030″ E
*Fagus sylvatica* L. forest of Sollazzotto	Typical montane mesothermal beech forest/Mesophilous beech forest on siliceous substrates	5.63	37°53′28.727″ N 14°32′44.805″ E
Bosco di Malabotta	Typical montane mesothermal beech forest, typical Turkey oak forest on mesoxeric soils/Mesophilous beech forest on siliceous substrates, montane Turkey oak forest	115.22	37°57′40.102″ N 15°3′17.785″ E
*Fagus sylvatica* L. forest of Serra del Re	Typical montane mesothermal beech forest/Mesophilous beech forest on siliceous substrates	14.34	37°56′39.617″ N 14°47′16.552″ E
*Fagus sylvatica* L. forest of Foresta Vecchia	Typical montane mesothermal beech forest/Mesophilous beech forest on siliceous substrates	10.03	37°56′5.809″ N 14°47′52.558″ E
*Fagus sylvatica* L. forest of Grappidà	Typical montane mesothermal beech forest/Mesophilous beech forest on siliceous substrates	10.60	37°55′56.186″ N 14°46′49.959″ E
*Quercus cerris* L. forest of Semantile	Typical Turkey oak forest on mesoxeric soils/Montane Turkey oak forest	83.71	37°53′26.117″ N 14°45′25.572″ E
*Fagus sylvatica* L. forest of Fago Scuro	Typical montane mesothermal beech forest/Mesophilous beech forest on siliceous substrates	8.31	37°56′49.936″ N 14°54′8.420″ E
*Pinus nigra* Arnold subsp. *calabrica* (Land.) E. Murray and *Quercus pubescens* Willd. s.l. forest of Monte Egitto	Typical montane *Pinus nigra* subsp. *calabrica* forest, downy oak forest on siliceous substrates, trembling aspen stand/Upper *Pinus nigra* subsp. *calabrica* forest, trembling aspen stand, downy oak forest on siliceous substrates (particularly *Quercus congesta* C. Presl)	3.11	37°45′52.874″ N 14°55′38.758″ E
*Fagus sylvatica* L. forest of Monte Spagnolo	Typical montane mesothermal beech forest/Beech forest on lava substrates of Etna	22.20	37°49′27.003″ N 14°57′45.264″ E
*Pinus nigra* subsp. *calabrica* forest of Ragabo	Typical montane *Pinus nigra* subsp. *calabrica* forest/Lower *Pinus nigra* subsp. *calabrica* forest	77.55	37°48′28.631″ N 15°4′9.534″ E
*Quercus suber* L. forest of Capotumino	Cork oak forest on xeric soils with maquis species/Cork oak forest on Iblean volcanic substrates	6.99	37°10′51.941″ N 14°53′30.006″ E

**Table 2:** Numerical representation of bryophyte taxa known to date in the forests proposed for inclusion in the Italian Network of old-growth forests.

Taxa tot.	Liverworts	Mosses	
183	28	155	**n. taxa**
101	20	81	**n. genera**
51	17	34	**n. families**

## Data Availability

The original contributions presented in this study are included in the article/Supplementary Materials. Further inquiries can be directed to the corresponding author.

## Supplementary Materials

The following supporting information can be downloaded at https://www.mdpi.com/article/10.3390/plants14182824/s1, Table S1: List of taxa found in Sicilian forests with old-growth traits and their threat categories.
